# Erratum to: Volume 1, Contraception and Reproductive Medicine

**DOI:** 10.1186/s40834-016-0017-2

**Published:** 2016-04-14

**Authors:** Contraception and Reproductive Medicine

**Affiliations:** BioMed Central, Floor 6, 236 Gray’s Inn Road, London, WC1X 8HB UK

Due to a technical issue with the set-up of the journal *Contraception and Reproductive Medicine* in production, the articles [1–5] were published with the incorrect volume and year information in the PDF of the article. In the HTML versions of the articles [1–5], the articles were published with incorrect volume information. The articles [1–5] were published into volume 2 and year 2017; however they should have been published in volume 1 and year 2016.

Each article [1–5] has since been updated with the correct volume and year information and is also listed with the correct information in the references in this erratum.

The publisher takes full responsibility for this error and sincerely apologises for the inconvenience caused.
